# Light-triggered toll-like receptor activation in a nanoscale metal–organic framework for synergistic PDT and cancer immunotherapy

**DOI:** 10.1039/d5sc03446a

**Published:** 2025-08-06

**Authors:** Yibin Mao, Langston Tillman, Xiaomin Jiang, Wangqing Bian, Chaoyu Wang, Tobias Fromme, Ralph R. Weichselbaum, Wenbin Lin

**Affiliations:** a Department of Chemistry, The University of Chicago Chicago Illinois 60637 USA wenbinlin@uchicago.edu; b Pritzker School of Molecular Engineering, The University of Chicago Chicago Illinois 60637 USA; c Department of Radiation and Cellular Oncology and Ludwig Center for Metastasis Research, The University of Chicago Chicago Illinois 60637 USA; d Chair of Molecular Nutritional Medicine, TUM School of Life Sciences, Technical University of Munich Freising Germany; e EKFZ – Else Kröner Fresenius Center for Nutritional Medicine, Technical University of Munich Freising Germany

## Abstract

Although innate immune modulators (IIMs) have shown promise as cancer immunotherapeutics, their clinical application is hindered by the challenge of achieving tumour-specific activation while minimizing systemic immune-related toxicity. Nanoscale metal–organic frameworks (MOFs) have emerged as effective carriers for photosensitizers to enable photodynamic therapy (PDT), which induces immunogenic cell death *via* reactive oxygen species (ROS) generation. We hypothesized that covalent conjugation of IMMs to nanoscale MOFs through ROS-cleavable linkers could localize immune activation to the tumour microenvironment while synergizing with PDT to enhance antitumour immunity. Here, we report the design of a hafnium-based nanoscale MOF, Hf-QP-DBP (QP and DBP denote amino-quaterphenyl dicarboxylate and 5,15-di(*p*-benzoato)-porphyrin ligands, respectively), functionalized with Resiquimod (R848) *via* a ROS-sensitive linker for combined PDT and immunotherapy. Upon light irradiation, the DBP photosensitizer within the MOF generates both singlet oxygen and hydroxyl radicals, enabling simultaneous induction of PDT and triggered release of R848. This dual-function platform effectively induces cancer cell death and suppresses tumour growth in colon cancer models, demonstrating its potential as an on-demand and synergistic cancer immunotherapy strategy.

## Abstract 2

A MOF with photosensitizers and TLR agonists synergized PDT and immune activation. Upon irradiation, it generates ROS to induce immunogenic cell death and release TLR agonists to enhance DC maturation, T-cell infiltration, and antitumour immunity.

## Graphical abstract

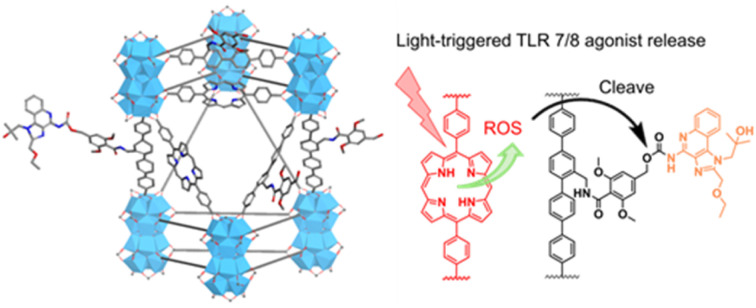

## Introduction

Over the past two decades, metal–organic frameworks (MOFs) have emerged as a versatile class of porous molecular materials with potential for a variety of applications, including gas storage, separation, and heterogeneous catalysis.^1–7^ By leveraging their inherent synthetic flexibility through the incorporation of appropriate molecular components, nanoscale MOFs have been tailored for many biomedical applications such as magnetic resonance imaging, X-ray computed tomography, and anticancer therapy.^8–10^ In particular, MOFs have been investigated as drug carriers by directly loading therapeutics into their pores, coordinating drug molecules to secondary building units (SBUs), or covalently attaching drugs to the organic linkers.^11–14^ However, while physisorption and coordination approaches often suffer from premature drug release, covalent attachment generally requires a specific stimulus to trigger release within the target tissue.^15–17^

MOFs have also demonstrated significant potential in photodynamic therapy (PDT), offering high loading of photosensitizers with spatial separation that minimizes excited-state quenching and enables efficient generation of reactive oxygen species (ROS).^18–20^ MOF-mediated PDT can induce immunogenic cell death (ICD), resulting in the release of danger-associated molecular patterns (DAMPs) and tumour-associated antigens (TAAs).^21–23^ Nonetheless, tumour resistance to PDT can emerge due to the development of an immunosuppressive tumour microenvironment (TME), ultimately limiting therapeutic efficacy.

A promising approach to overcome this limitation involves combining PDT with innate immune modulators (IIMs) such as Toll-like receptor (TLR) agonists to amplify antitumour immune responses. TLR agonists like Resiquimod (R848) activate antigen-presenting cells, stimulating the production of proinflammatory cytokines such as IL-6 and TNF-α, thereby enhancing antigen presentation and immune cell infiltration.^24,25^ Although R848 has demonstrated strong therapeutic potential in preclinical studies, its systemic use is limited by significant side effects.^26,27^ Conversely, its clinical efficacy as a topical agent for cutaneous malignancies highlights its potential when delivered locally to limit systemic exposure.^28,29^

To address these challenges, we propose incorporating R848 into a photosensitizing MOF *via* a ROS-responsive linker to achieve light-triggered release of the immune agonist within the TME during PDT. We hypothesize that the DAMPs and TAAs released through PDT-induced ICD will synergize with TLR7/8 activation by R848 to elicit robust anti-tumour immune responses. Although previous studies have demonstrated synergism between PDT and TLR agonists,^30,31^ our platform uniquely enables spatially controlled release of R848 *via* light-induced cleavage, reducing the risk of systemic toxicity from off-target immune activation.

**Fig. 1:**
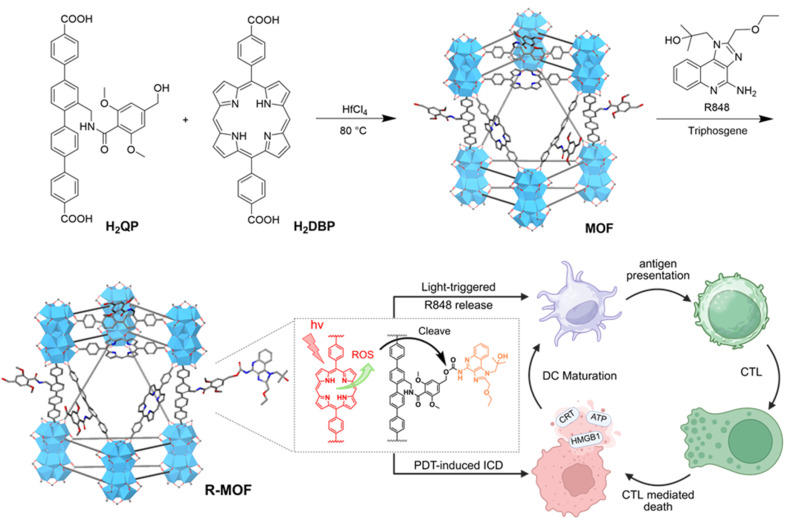
Synthesis of Hf-DBP-QP (MOF) and its covalent conjugation with R848 *via* a ROS-cleavable linker to yield R-MOF, a dual-function platform that mediates PDT and immune activation. Upon light irradiation, R-MOF induces ICD and locally releases R848, promoting dendritic cell maturation and T cell infiltration within the TME.

Herein, we report the design of a photosensitizing nanoscale MOF incorporating amino-quaterphenyl dicarboxylate (QP) and 5,15-di(*p*-benzoato)-porphyrin (DBP) mixed ligands, and its covalent functionalization to R848 *via* a ROS-responsive 3,5-dimethoxybenzyl carbamate linker (termed MOF and R-MOF, respectively) for synergistic PDT and immune activation (Fig. 1).^32–34^ Hafnium is a tetravalent transition metal and a hard Lewis acid that forms strong coordination bonds with carboxylate-based bridging ligands, contributing to the exceptional chemical and structural stability of hafnium-based MOFs. The thermodynamic preference of Hf^4+^ for forming stable HfO_2_ reduces the likelihood of its coordination to proteins or other biomolecules, lowering the risk of long-term toxicity. Importantly, Hf-based MOFs were the first to enter human clinical trials,^35^ supporting their excellent biosafety, biocompatibility, and translational potential in biomedical applications. The DBP ligand is a hydrophobic photosensitizer that promotes ROS generation and structural stability. The QP ligand was selected for its comparable molecular length to DBP, ensuring structural compatibility within the mixed-ligand framework, and its ability to be modified with a ROS-sensitive linker. Upon light irradiation, R-MOF mediates PDT-induced tumour cell death and simultaneously releases R848 locally within the TME. This dual action promotes the release of DAMPs and TAAs, enhances antigen presentation, and stimulates robust innate and adaptive antitumour immune responses, leading to significant tumour regression in two murine colorectal cancer models.

## Results and discussion

### Synthesis and characterization of R-MOF

The porphyrin ligand H_2_DBP was synthesized following previously reported procedures.^36^ The quaterphenyl dicarboxylic acid (H_2_QP) with a pendant 3,5-dimethylbenzyl alcohol group was synthesized according to Scheme S1.^37^ The mixed-ligand nanoscale MOF was synthesized by heating a mixture of HfCl_4_, H_2_DBP, H_2_QP, acetic acid (AcOH), and water at 80 °C for 2 days. R848 was covalently attached to the QP ligands in the MOF to form R-MOF through the formation of a carbamate bond with the assistance of triphosgene. To validate the post-synthetic modification strategy, a molecular analog, Me_2_QP-R848, was synthesized from Me_2_QP-OH following the same route.

**Fig. 2:**
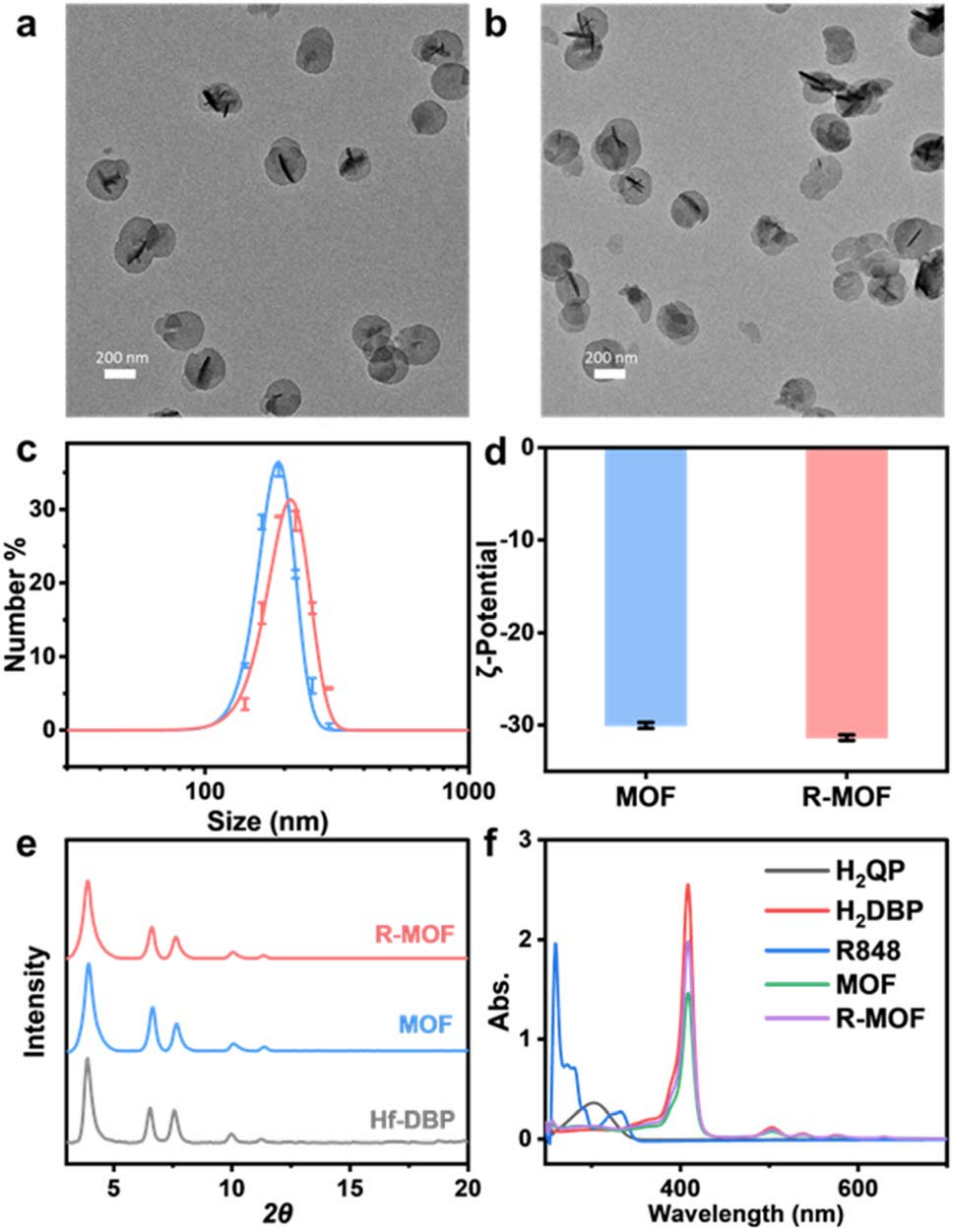
TEM images of (a) MOF and (b) R-MOF. Scale bar: 200 nm. (c) Number-averaged sizes of MOF and R-MOF. (d) Zeta (*ζ*) potentials of MOF and R-MOF. (e) PXRD patterns of R-MOF, MOF, and Hf-DBP. (f) UV-vis spectra of digested MOF and R-MOF along with H_2_QP, H_2_DBP, and R848 in dimethyl sulfoxide.

Transmission electron microscopy (TEM) revealed that the MOF exhibited a nanoplate morphology with a diameter of approximately 200 nm and a thickness of ∼20 nm (Fig. 2a). After R848 conjugation, R-MOF maintained the nanoplate morphology with no noticeable change in particle size (Fig. 2b). The number-averaged sizes of MOF and R-MOF were measured to be 157 ± 6 nm and 165 ± 6 nm, respectively (Fig. 2c), while their *ζ*-potentials were measured as −30.03 ± 0.32 mV and −31.37 ± 0.30 mV by dynamic light scattering (Fig. 2d). Powder X-ray diffraction (PXRD) analysis revealed that both MOF and R-MOF exhibited similar patterns to Hf-DBP,^36^ consistent with the Hf_12_ MOF structure consisting of Hf_12_(μ_3_-O)_8_(μ_3_-OH)_8_(μ_2_-OH)_6_ SBUs and linear dicarboxylate ligands arranged in an hcp topology (Fig. 2e).

^1^H NMR analysis of the digested MOF indicated a DBP/QP ratio of 2.7 and an AcOH/QP ratio of 2.3 based on the integration of the characteristic peaks for AcOH, H_2_QP, and H_2_DBP (Fig. S2). The observed DBP/QP ratio was consistent with the synthetic feed ratio (3.0). The concentration of DBP was quantified by comparing the UV-vis absorption peaks of the MOF digest to a standard calibration curve of H_2_DBP. These analyses revealed an empirical formula of Hf_12_(μ_3_-O)_8_(μ_3_-OH)_8_(μ_2_-OH)_6_(DBP)_5.0_(QP)_1.9_(AcO)_4.3_ for the MOF.

To quantify R848 loading, R-MOF was digested using sodium bicarbonate, and the carbamate linkage was hydrolysed with sodium hydroxide. The resulting solution was analysed by liquid chromatography-mass spectrometry (LC-MS). These analyses showed that approximately 88% of QP ligands in R-MOF were successfully conjugated with R848, while ∼1.2% of the total R848 was non-covalently trapped in the R-MOF pores. Accordingly, the empirical formula for R-MOF was determined to be Hf_12_(μ_3_-O)_8_(μ_3_-OH)_8_(μ_2_-OH)_6_(DBP)_5.0_(QP-R848)_1.7_(QP)_0.2_(AcO)_4.3_(R848)_0.02_.

### R-MOF produces ROS and cleaves R848 to stimulate dendritic cells

**Fig. 3:**
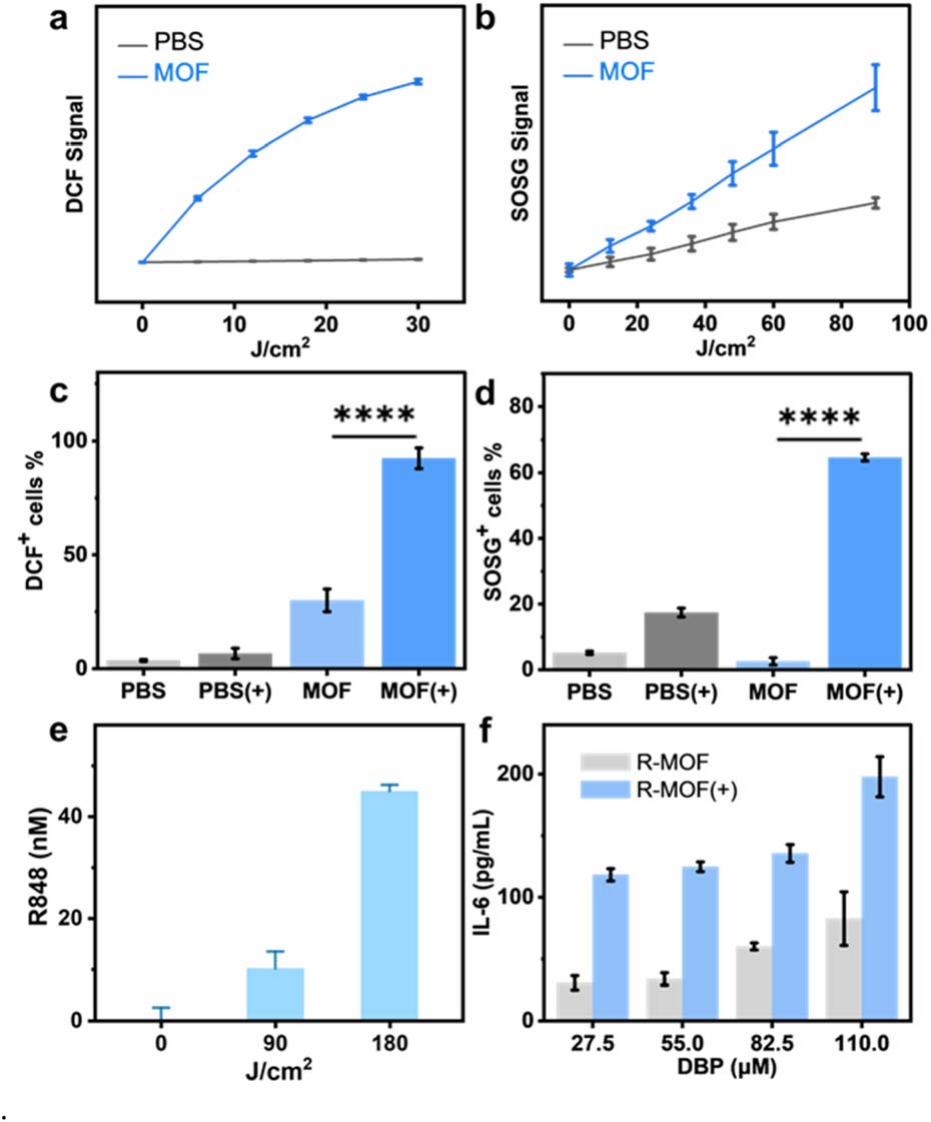
(a) Total ROS generation by the DCF assay and (b) ^1^O_2_ generation by the SOSG assay in test tubes, *n* = 3. Flow Cytometry of CT26 cells showing (c) DCF-positive cells and (d) SOSG-positive cells, *n* = 3. (e) Power-dependent PDT-induced R848 release as analysed by LC-MS. (f) IL-6 secretion by BMDCs after indicated treatments, as detected by ELISA. ****P < 0.0001.

We first evaluated the ability of the MOF to generate total ROS and singlet oxygen using the 2′,7′-dichlorodihydrofluorescein (DCF) assay and the singlet oxygen sensor green (SOSG) assay, respectively. Under 630 nm light irradiation and a DBP concentration of 5 mM, the MOF [denoted MOF(+)] enhanced total ROS and singlet oxygen (^1^O_2_) generation by 45-fold and 2-fold over PBS, respectively (Fig. 3a and b). The release of R848 upon PDT treatment was evaluated by LC-MS, showing that R-MOF at a DBP concentration of 60 mM released 45 nM R848 upon exposure to 180 J cm^−2^ of 630 nm light (Fig. 3e). The concentration of the released R848 is three-fold higher than the EC_50_ of R848 for TNF-α secretion by RAW264.7 macrophages (Fig. S4).

To evaluate intracellular ROS generation, we conducted DCF staining of CT26 cells followed by flow cytometry analysis. MOF(+) treatment at a DBP concentration of 20 mM induced strong intracellular ROS production, with 84% and 80% cells staining DCF positive, respectively, compared to ∼34% in the PBS plus light irradiation [denoted PBS(+)] group (Fig. 3c and S5). We further investigated the specific ROS generated by the MOF. Intracellular production of hydroxyl radicals and ^1^O_2_ in CT26 cells was evaluated by hydroxyphenyl fluorescein (HPF) and SOSG staining, respectively. Flow cytometry analysis revealed a 192-fold increase in HPF-positive CT26 cells and a 25-fold increase in SOSG-positive CT26 cells treated with MOF(+) compared to the MOF group, indicating the generation of both hydroxyl radicals and ^1^O_2_ by MOF(+) (Fig. 3d and S6). The intracellular hydroxyl radical production was confirmed by CLSM imaging, which revealed a substantial increase in HPF fluorescence by MOF(+) treatment compared to MOF alone and PBS(+) groups (Fig. S5). These results demonstrate the ability of MOF(+) in generating both hydroxyl radicals and ^1^O_2_ for triggering R848 release and inducing cytotoxicity.

Having established light-activated R848 release from R-MOF, we next assessed its immunostimulatory effect on dendritic cells. IL-6, a key cytokine involved in dendritic cell differentiation and activation,^38^ was significantly elevated in bone marrow-derived dendritic cell (BMDC) cultures treated with R-MOF plus light irradiation [denoted R-MOF(+)] at a DBP concentration of 27.5–110 μM, compared to R-MOF alone, with a 3.8-fold increase observed at 27.5 μM DBP (Fig. 3f). Similarly, the levels of TNF-α, another pro-inflammatory cytokine,^39^ were significantly higher in BMDCs treated with R-MOF(+), showing a 38% increase in secretion compared to R-MOF alone at the same R848 concentration (Fig. S7).

### R-MOF undergoes cellular uptake and induces apoptosis upon light irradiation

**Fig. 4:**
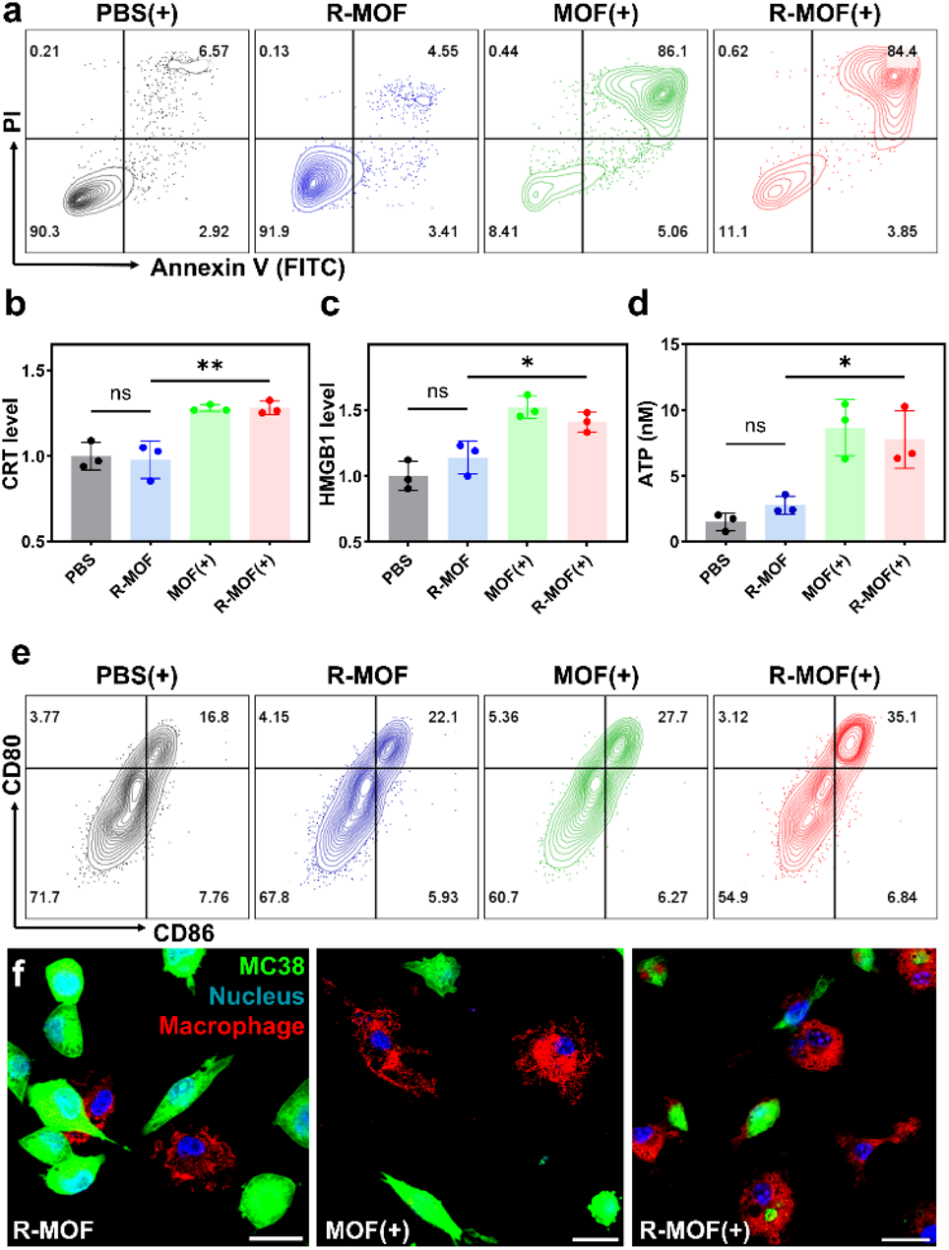
(a) Apoptosis analysis of CT26 cells following the indicated treatments. (b) CRT exposure, (c) HMGB1 release, and (d) extracellular ATP levels after the indicated treatments (*n* = 3). (e) BMDC maturation upon exposure to treated samples. (f) CLSM images showing phagocytosis of treated MC38 cells by BMDMs. Scale bar = 20 μm. *P < 0.05 and **P < 0.01.

To determine the cytotoxicity of MOF(+) on cancer cells, we first confirmed time-dependent cell uptake of the MOF. CT26 cells were co-incubated with MOF for different amounts of time, and the DBP content within the cells was detected *via* flow cytometry. The experiment demonstrated that around 95% of cells were DBP+ after 8 hours of incubation (Fig. S8). A cell viability assay demonstrated minimal dark toxicity of the MOF, while significant toxicity was observed upon light irradiation. The IC_50_ values of MOF(+) were 12.83 ± 8.67, 5.5 ± 1.2, and 21.55 ± 2.65 μM for MC38, CT26, and 4T1 cells, respectively (Fig. S9), indicating efficient cellular uptake of the MOF and ROS-mediated cytotoxicity upon light activation. Next, we analyzed the apoptotic pathway induced by PDT. Annexin V and PI staining revealed that approximately 85% of CT26 cells treated with MOF(+) or R-MOF(+) were in the late apoptotic stage (Fig. 4a).

### R-MOF-mediated PDT induces immunogenic cell death and stimulates innate immunity

We also measured key markers for ICD: calreticulin (CRT) translocation, extracellular ATP release, and high mobility group box 1 (HMGB1) secretion. Both MOF(+) and R-MOF(+) treatments triggered a ∼4-fold increase in extracellular ATP, a ∼30% increase in CRT exposure, and a ∼50% increase in HMGB1 release (Fig. 4b–d, S10), suggesting that ICD is primarily driven by PDT.

To assess the synergy between PDT-induced ICD and R848 release, we first evaluated dendritic cell maturation. Supernatants from treated CT26 cells were incubated with BMDCs from BALB/c mice to probe the combined effect of DAMPs and R848. Flow cytometry analysis of CD11b^+^ cells co-expressing CD80 and CD86 showed that while both R-MOF and MOF(+) increased mature dendritic cell (mDC) levels relative to the PBS(+) control (Fig. 4e and S11), R-MOF(+) induced significantly greater maturation (35.1%), more than doubling the rate observed in the PBS(+) group (16.8%). Similarly, supernatants from treated CT26 cells were incubated with RAW264.7 cells. The RAW264.7 cells in the R-MOF(+) treatment group exhibited a 3.5-fold increase of Phospho-NF-κB p65 over the MOF(+) treatment group, supporting the synergy between PDT and R848 (Fig. S12). We next investigated phagocytosis following treatment. MC38 cells were pre-labeled with CFSE, treated, and then co-cultured with bone marrow-derived macrophages (BMDMs) from C57BL/6 mice. Confocal microscopy revealed minimal phagocytic uptake in the R-MOF and MOF(+) groups, while R-MOF(+) treatment led to robust macrophage phagocytosis (Fig. 4f).

Collectively, these findings demonstrate that while PDT drives ICD and the release of danger signals, the light-triggered release of R848 from R-MOF enhances immune engagement by promoting both dendritic cell maturation and macrophage phagocytosis. These results support the superior immunostimulatory potential of R-MOF(+) over MOF(+) due to its ability to induce a more robust immune response.

### R-MOF elicits anti-tumour activity and induces T cell recruitment to the tumour

**Fig. 5:**
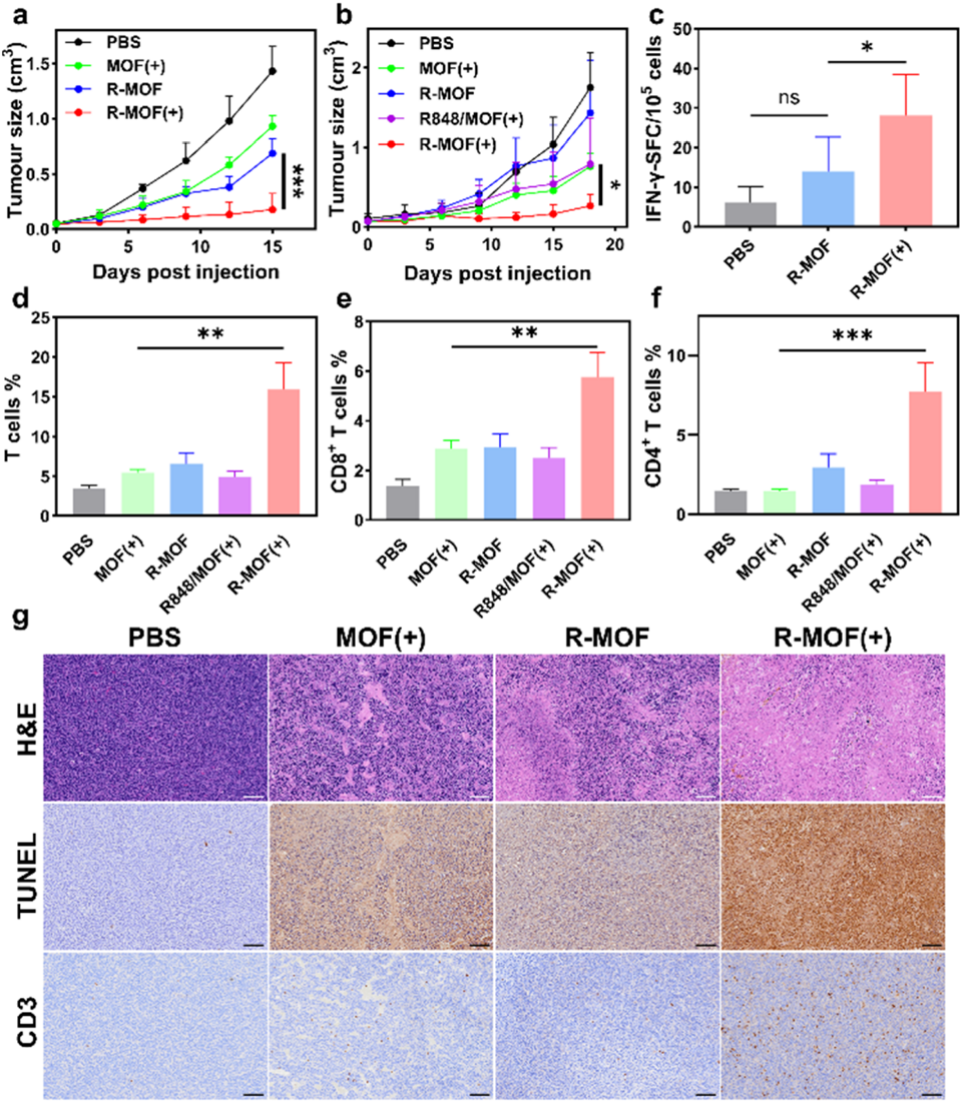
*In vivo* tumour growth curves of subcutaneous (a) CT26 and (b) MC38 models (*n* = 5). (c) IFN-γ ELISPOT analysis of splenocytes of treated MC38 tumour-bearing C57BL/6 mice (*n* = 5) in response to an MC38-derived antigen (KSPWFTTL). (d)–(f) T-cell analysis of excised MC38 tumours 14 days after treatment. (g) H&E, TUNEL, and CD3 staining of excised CT26 tumours 14 days after treatment. Scale bar = 100 μm. *P < 0.05, **P < 0.01, and ***P < 0.001.

To evaluate the *in vivo* efficacy of R-MOF, we established a subcutaneous CT26 tumour model. CT26-bearing mice received intratumoural injections of PBS, MOF, or R-MOF at a dose of 0.5 μmol DBP, followed by LED light irradiation (100 mW cm^−2^, 630 nm, 15 min) at the tumour site 8 hours post-injection. Tumour volumes and body weights were monitored daily. While both MOF(+) and R-MOF induced some degree of tumour suppression, R-MOF(+) achieved markedly superior therapeutic outcomes, with a tumour growth inhibition (TGI) of 87.6% and a complete response in 1 out of 5 mice (Fig. 5a).

To further validate these results, a subcutaneous MC38 tumour model was established. MC38-bearing mice were treated intratumourally with PBS, MOF, R-MOF, or MOF combined with QP-R848 at a DBP dose of 0.5 μmol and an equivalent R848 dose of 50 μg. This additional group was included to evaluate the additive effects of free R848 and PDT (Fig. 5b). Consistent with the CT26 findings, R-MOF(+) outperformed all other groups, achieving a TGI of 84.7%. In comparison, MOF(+) and R848 plus MOF(+) groups reached TGIs of 56.4% and 54.8%, respectively, while the R-MOF group showed limited efficacy (TGI = 18.3%).

Histological analysis of CT26 tumours revealed extensive cellular damage and nuclear depletion in the R-MOF(+) group, as shown by H&E staining. TUNEL staining confirmed increased DNA fragmentation, while immunohistochemical analysis demonstrated greater immune cell infiltration in R-MOF(+) tumours, with elevated levels of CD3^+^ T cells and IBA-1^+^ macrophages.

To investigate systemic immune activation, we analysed splenocytes from C57BL/6 mice in the MC38 model *via* an IFN-γ ELISpot assay. R-MOF(+) treatment significantly enhanced systemic immunity, doubling the number of IFN-γ-secreting spot-forming cells (SFCs) relative to R-MOF and more than quadrupling that of the PBS group (Fig. 5c and S13). This increase in systemic antitumour immunity correlated with elevated T-cell infiltration and enhanced T-cell differentiation in the R-MOF(+) group compared to controls (Fig. 5d–f and S14).

Consistent with flow cytometry data, histological staining of excised tumours revealed reduced nuclear density and increased DNA fragmentation post-treatment (Fig. 5g and S15). Immunostaining further confirmed enhanced CD3^+^ T-cell and IBA-1^+^ macrophage infiltration, alongside reduced tumour cell proliferation, as indicated by lower Ki-67 expression (Fig. 5g and S15).

Lastly, R-MOF(+) treatment exhibited no systemic toxicity. Major organ histology (Fig. S16) and stable body weights throughout the study (Fig. S17) support the biocompatibility of the treatment. Furthermore, blood samples taken from treated mice were tested 48 hours after treatment and demonstrated no increase in creatinine or AST activity, indicating a lack of hepatotoxicity and nephrotoxicity (Fig. S18). Taken together, R-MOF(+) effectively suppresses tumour growth and elicits strong systemic antitumour immunity by promoting dendritic cell activation, enhancing T-cell infiltration, and fostering macrophage involvement.

## Conclusions

In this work, we report the development of a drug-conjugated MOF that enables the light-triggered release of the TLR7/8 agonist R848 during PDT. This rational design integrates PDT with immunotherapy to overcome longstanding challenges associated with the delivery and efficacy of innate immune agonists. By leveraging the release of R848 in response to ROS, our platform synergistically combines PDT-induced immunogenic cell death with localized TLR activation. This strategy fosters a TME conducive to immune cell infiltration, promotes dendritic cell maturation, and amplifies antitumour immune responses. Our findings highlight the potential of MOF-based delivery systems for spatiotemporally controlled release of immunomodulators and provide a strong rationale for combining PDT with immune-stimulating agents to enhance therapeutic efficacy.

## Author contributions

X. Jiang and W. Lin conceived the project. Y. Mao, L. Tillman, X. Jiang, C. Wang, and W. Bian performed the experiments and analysed the results. Y. Mao, L. Tillman, X. Jiang, T. Fromme, R. R. Weichselbaum, and W. Lin wrote the manuscript.

## Conflicts of interest

There are no conflicts to declare.

## Supplementary Material

SC-016-D5SC03446A-s001

## Data Availability

The datasets supporting this article have been uploaded as part of the supplementary information. Supplementary information is available: synthesis and characterization, *in vitro* studies, and *in vivo* studies. See DOI: https://doi.org/10.1039/d5sc03446a.
