# Supplementary material for: Light-triggered toll-like receptor activation in a nanoscale metal–organic framework for synergistic PDT and cancer immunotherapy
Source: Chem Sci. 2025 Aug 6;16(35):16314–20. doi: 10.1039/d5sc03446a (PMC12355220; doi:10.1039/d5sc03446a)
Supplement: SC-016-D5SC03446A-s001 [file SC-016-D5SC03446A-s001.pdf]

Supplementary Information (SI) for Chemical Science.
This journal is © The Royal Society of Chemistry 2025

Supporting Information for

A Nanoscale Metal–Organic Framework with Light-Triggered Toll-Like Receptor Activation for Synergistic PDT and Immunotherapy

Yibin Mao,[†,a] Langston Tillman,[†, b] Xiaomin Jiang,[a,c] Wangqing Bian,[a,d] Chaoyu Wang,[a,c] Tobias Fromme,[d,e] Ralph R. Weichselbaum,[c] Wenbin Lin*[,a,c]

[a]Department of Chemistry, The University of Chicago, Chicago, Illinois 60637, United States

[b]Pritzker School of Molecular Engineering, The University of Chicago, Chicago, Illinois 60637, United States

[c]Department of Radiation and Cellular Oncology and Ludwig Center for Metastasis Research, The University of Chicago, Chicago, Illinois 60637, United States

[d]Chair of Molecular Nutritional Medicine, TUM School of Life Sciences, Technical University of Munich, Freising, Germany

[e]EKFZ - Else Kröner Fresenius Center for Nutritional Medicine, Technical University of Munich, Freising, Germany

KEYWORDS: Metal-organic frameworks • toll-like receptor • immunotherapy • photodynamic therapy • triggered release

S1 Experimental Section

S1.1 Materials

All starting materials were purchased from Sigma-Aldrich and ThermoFisher (USA) unless otherwise noted and used without further purification. Transmission electron microscopy (TEM) was carried out on a TECNAI Spirit and a TECNAI F30 HRTEM. Powder X-ray diffraction (PXRD) data was collected on a Bruker D8 Venture diffractometer using a Cu Kα radiation source (λ = 1.54178 Å) and processed with PowderX software. UV-vis spectra were collected using a Shimadzu UV-2600 UV-vis spectrophotometer. Dynamic light scattering (DLS) and ζ potential measurements were performed on a Malvern Zetasizer Nano ZS instrument. ^{1}H NMR spectra were recorded on a Bruker NMR 400 DRX spectrometer at 400 MHz and referenced to the proton resonance resulting from incomplete deuteration of $CDCl_3$ (δ = 7.26) or DMSO-d_6 (δ = 2.50). Flow cytometry data was collected on an LSR-Fortessa 4-15 (BD Biosciences, USA) and analyzed by FlowJo software (Tree Star, USA). Confocal laser scanning microscopy (CLSM) images were collected on a Leica Stellaris 8 laser scanning confocal microscope at the University of Chicago Integrated Light Microscopy Facility and analyzed with ImageJ software (NIH, USA). The histological slides were scanned on a CRi Pannoramic SCAN 20x whole slide scanner by Integrated Light Microscopy Core in the University of Chicago and analyzed with the

QuPath-0.2.3 software. The absorbance and fluorescence signals from well plates were read by a BioTek Synergy HTX microplate reader.

PBS was purchased from ThermoFisher. Trypsin-EDTA solution was purchased from the American Type Culture Collection (ATCC, Rockville, MD). 3-(4,5-Dimethylthiazol-2-yl)-5-(3-carboxymethoxyphenyl)-2-(4-sulfo-phenyl)2H-tetrazolium (MTS) was purchased from Promega (USA). Murine colorectal carcinomas lines CT26 and MC38 cells and murine triple negative breast cancer cell line 4T1 were purchased from ATCC. CT26 and 4T1 cells were cultured in RPMI-1640 (Corning, USA) while MC38 cells were cultured in DMEM (Gibco, USA). RPMI-1640 and DMEM media were supported with 10% fetal bovine serum (VWR, USA), 100 U/ml penicillin G sodium, and 100 µg/ml streptomycin sulfate. The cells were kept in a humidified atmosphere containing 5% CO_2 at 37°C. BALB/c breeders were obtained from Charles River Laboratories (USA) and bred in-house at the animal facility at the University of Chicago. BALB/c mice with an age of 6-8 weeks were used for in vivo experiments. The study protocol was reviewed and approved by the Institutional Animal Care and Use Committee (IACUC) at the University of Chicago (PHS Assurance #D16-00322 (A3523-01)). The Human Tissue Resource Center at the University of Chicago provided histology related services for this study.

S1.2. Synthesis of Molecules and Materials

H_2QP and H_2DBP were synthesized according to previously reported procedures.[1,2]

Synthesis of Hf-DBP-QP (MOF)

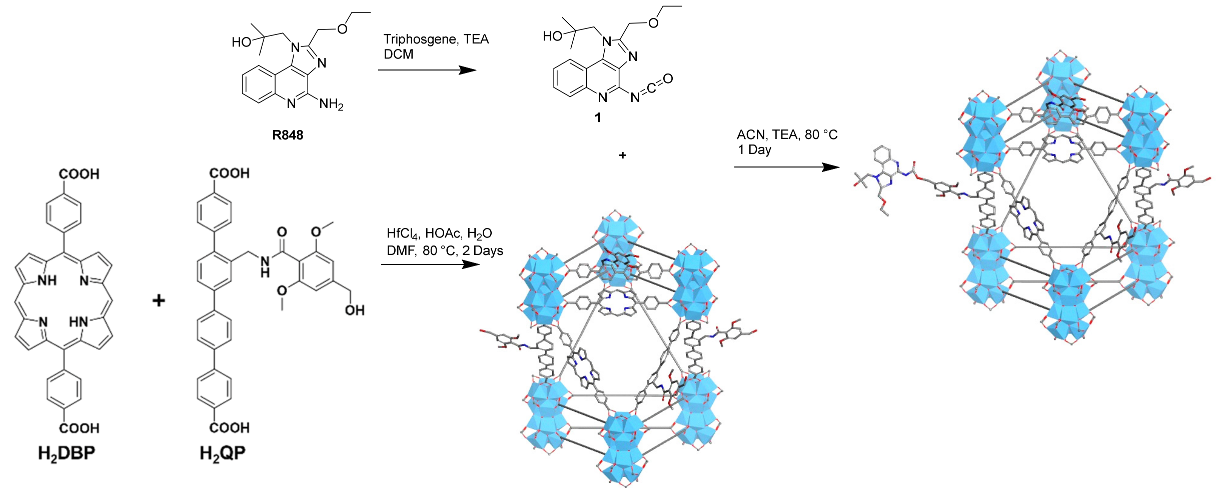

Scheme S1. Synthesis of MOF and R-MOF

$HfCl_4$, H_2DBP, and H_2QP were separately dissolved in degassed DMF at a concentration of 2, 3.5, and 3.5 mg/mL, respectively. 30 mL $HfCl_4$ solution, 21.9 mL H_2DBP solution, and 8.1 mL H_2QP solution were then combined with the addition of 0.3 mL H_2O and 3 mL acetic acid. The mixture was sealed under N_2 and stirred at 80 °C for 48 hours. The purple precipitate was collected by centrifugation and sequentially washed with DMF, 1% TEA in ethanol (EtOH) (v/v), and EtOH, and then dispersed in 60 mL EtOH for storage.

Synthesis of Hf-DBP-QP-R848 (R-MOF)

R848 (240 mg, 0.76 mmol) and TEA (300 µL) were dissolved in dry DCM (40 mL), and the resulting solution was cooled to -78 °C. In a separate vial, triphosgene (120 mg, 0.4 mmol) was dissolved in 10 mL of dry DCM and then added dropwise to the R848 solution under stirring. The resulting mixture was warmed to room temperature and stirred for 2 hours. The mixture was concentrated under reduced pressure to afford the crude product of 1-(2-(ethoxymethyl)-4-isocyanato-1H-imidazo[4,5-c]quinolin-1-yl)-2-methylpropan-2-ol (**10)**, which is used in next step without further purification.

187 mL of MOF dispersion in EtOH was washed with ACN twice and dry ACN twice and dispersed in dry ACN. The resulting mixture was added to the crude product of **1**, and the mixture was stirred at room temperature overnight. The mixture was then centrifuged, washed with ACN 10 times, and the resulting R-MOF was dispersed in ACN for storage.

Synthesis of Me_2QP-R848

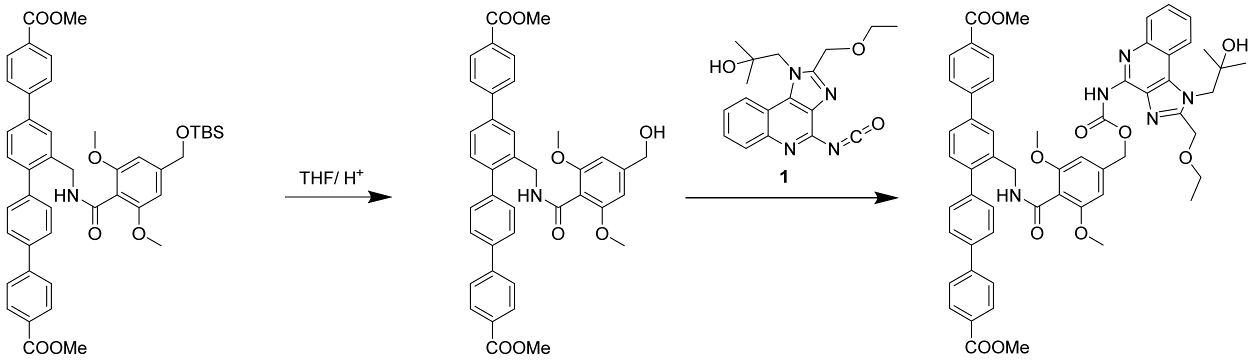

2 3 **Me_2QP-R848**

Scheme S2. Synthesis of Me2QP-R848

To a solution of compound **2** (100 mg, 0.131 mmol) in THF (5 mL) was added 100 µL 1 M HCl. The mixture was stirred at room temperature for 5 minutes and then concentrated under reduced pressure to afford the crude product Me_2QP-OH (**3**), which was dissolved in 10 mL of ACN before the addition of the crude product of compound **1** (80 mg). The resulting mixture was stirred at room temperature overnight and then concentrated under reduced pressure. The residue was purified using silica gel column chromatography (DCM/MeOH = 30:1) to yield Me_2QP-R848 (98 mg, 0.099 mmol, 76.0%). 1H NMR (400 MHz, CDCl3) δ 8.16 (dd, J = 8.5, 7.0 Hz, 5H), 8.01 (d, J = 1.9 Hz, 1H), 7.80 – 7.72 (m, 6H), 7.64 (dd, J = 7.9, 2.0 Hz, 2H), 7.57 – 7.49 (m, 3H), 7.43 (d, J = 7.9 Hz, 1H), 6.67 (s, 2H), 5.29 (s, 2H), 4.93 (s, 2H), 4.85 – 4.74 (m, 4H), 3.98 (d, J = 2.9 Hz, 6H), 3.76 (s, 6H), 3.67 (p, J = 7.3 Hz, 4H), 1.45 – 1.33 (m, 9H). LC-MS (ESI, positive mode): *m/z* calc'd for $C_{55}H_{56}N_5O_{11}$ $[M+H]^-$: 986.3976, found 986.3977.

S1.3. Characterization of Molecules and Materials

MOF Digestion for UV-Vis Spectroscopic and ^{1}H NMR Measurements

50 µL MOF or R-MOF dispersion, 900 µL DMSO, and 50 µL H_3PO_4 were mixed and sonicated for 20 minutes. The mixture was diluted to a proper concentration for UV-vis measurement. The

absorption of H_2DBP at 408 nm was used to calculate the concentration of H_2DBP in Hf-DBP-QP or R-MOF after comparison with the standard curve of H_2DBP in DMSO.

2 mL MOF or R-MOF dispersion was centrifuged and the solid was dried in vacuum and dispersed in 900 µL D_6-DMSO, 50 µL D_3PO_4, and 50 µL D_2O. The mixture was sonicated for 20 minutes before ^1H NMR analysis. The integration ratio for the singlet at around δ10.67 and the doublet at δ7.55-7.57 was used to determine the ratio between H_2DBP and H_2QP.

Stability of R-MOF in PBS

R-MOF was dispersed in 1 mL PBS (1 mM) with a Hf concentration of 10 mM and was incubated at 37 °C for 24 hours. The solid was isolated by centrifugation for PXRD measurement.

Digestion of R-MOF for Free R848 Quantification by LC-MS

100 µL 1 M $NaHCO_3$ solution was added to 100 µL R-MOF dispersion in PBS. The mixture was sealed and sonicated for 30 minutes. The supernatant after centrifugation was analyzed by LC-MS.

Light Triggered R848 Release

R-MOF at an equivalent R848 concentration of 100 µM was dispersed in PBS (10 mM, pH 7.4). 110 µL R-MOF suspension was irradiated with red light with the wavelength of 630 nm at a power density of 50 mW/cm^2 for up to 60 minutes. 100 µL of the suspension was digested as above for the quantification of free R848 by LC-MS.

S1.4. In Vitro Studies

***In vitro* Cytotoxicity**

The cytotoxicity of MOF, R-MOF, and R848 in CT26, 4T1, and MC38 cells was evaluated by the 3-(4,5-dimethylthiazol-2-yl)-5-(3-carboxymethoxyphenyl)-2-(4-sulfophenyl)-2H-tetrazolium (MTS) assay. The cells were seeded in 96-well plates at a density of 5000 cells/well. Different concentrations of MOF, R-MOF, and R-848 were added and incubated for 8 hours. The wells were irradiated with LED light (630 nm, 80 mW/cm^{2,} 12.5 minutes). Twenty-four hours later, 10% (V/V) of MTS reagent was added to each well. Two hours later, the absorbance of each well at 490 nm was read by a Synergy HTX plate reader to calculate cell viability. IC_{50} values for each treatment group were calculated by fitting non-linear regression curves using GraphPad software.

***In vitro* ROS Generation**

To evaluate ROS generation, CT26 cells were seeded in culture dishes at a density of 2 × 10^5 cells per well and incubated overnight. MOF or R-MOF was then added at a DBP concentration of 20 µM, followed by incubation at 37 °C for 6 hours. For total ROS and hydroxyl radical detection, cells were washed three times with PBS and incubated with 1 mL of culture medium containing either 40 µM DCFH-DA (for total ROS) or 20 µM HPF (for hydroxyl radicals) at 37 °C for 30 minutes. Subsequently, cells were irradiated with LED light (630 nm, 80 mW/cm^2, 12.5

minutes), harvested by scraping, and analyzed by flow cytometry using the FITC channel. For $^1O_2$ detection, cells were first washed with DPBS containing 1% HyClone penicillin-streptomycin (100×) and incubated in this solution for 2 hours. Afterward, cells were washed with DPBS and incubated with 50 μM Singlet Oxygen Sensor Green (SOSG) for 30 minutes. Following three washes with DPBS, cells were irradiated with LED light (630 nm, 80 mW/cm², 12.5 minutes), harvested, and analyzed by flow cytometry (FITC channel).

For confocal laser scanning microscopy (CLSM) imaging, CT26 cells were seeded in 35 mm glass-bottom dishes (CellVis) at a density of 1 × 10^5 cells per dish and cultured overnight. MOF or R-MOF was then added at a DBP concentration of 20 μM and incubated at 37 °C for 6 hours. After incubation, the cells were washed three times with PBS and incubated with 1 mL of culture medium containing either 40 μM DCFH-DA (for total ROS detection) or 40 μM HPF (for hydroxyl radical detection) at 37 °C for 30 minutes. Cells were then irradiated with LED light (630 nm, 80 mW/cm², 12.5 minutes), washed three times with PBS, stained with Hoechst 33342, and imaged using a Leica Stellaris 8 Laser Scanning Confocal Microscope.

Apoptosis

To quantify apoptosis, CT26 cells were seeded in 6-well plates at a density of 2 × 10^5 cells per well and cultured overnight. MOF or R-MOF was then added at an equivalent DBP concentration of 20 μM and incubated for 6 hours, followed by LED irradiation (630 nm, 80 mW/cm², 12.5 minutes). After 24 hours, the cells were washed with PBS, trypsinized to obtain single-cell suspensions, and stained using the Dead Cell Apoptosis Kit with Annexin V Alexa Fluor 488 and PI, according to the manufacturer's protocol. The cells were then resuspended in binding buffer and analyzed by flow cytometry (Annexin V detected in the FITC channel; PI in the PE-Dazzle 594 channel).

Immunogenic Cell Death

CT26 cells were seeded in 6-well plates at a density of 2 × 10^5 cells per well and cultured overnight. MOF or R-MOF was then added at an equivalent DBP concentration of 20 μM. After 6 hours of incubation, cells in the irradiated groups were exposed to LED light (630 nm, 80 mW/cm², 12.5 minutes). Following an additional 24-hour incubation, the medium was removed, and cells were washed with PBS and trypsinized to obtain single-cell suspensions. For flow cytometry calreticulin (CRT) surface expression analysis, cells were stained with Alexa Fluor 488-labeled anti-calreticulin antibodies (1:150 dilution) in 1% BSA PBS solution (FACS buffer) on ice for 30 minutes. After staining, cells were washed with PBS, resuspended in FACS buffer, and analyzed by flow cytometry. For CLSM CRT imaging, cells were cultured and seeded in 35 mm Glass bottom dishes (CellVis) at a density of 1×10^5 cells/well and cultured overnight. The cells were then treated as induced. Following an additional 24-hour incubation, the medium was removed, the dishes were rinsed once with ice-cold PBS, then fixed with methanol at -20°C for five minutes. The cells were then stained with Alexa Fluor 488-labeled anti-calreticulin antibodies (1:200 dilution) in 1% BSA PBS solution for 30 minutes at room temperature. The dishes were then washed three times with PBS, stained with Hoechst 33342, and examined with CLSM.

For extracellular ATP and HMGB1 release assays, CT26 cells were seeded in 96-well plates at a density of 7,500 cells per well and incubated overnight in FBS-free RPMI-1640 medium. MOF or R-MOF was added to yield a DBP-equivalent concentration of 20 μM. After 8 hours of incubation at 37 °C, half of the wells were irradiated with LED light (630 nm, 80 mW/cm², 12.5 minutes). Cells were further incubated for 24 hours. The plates were then centrifuged (300 × g, 5 minutes), and the supernatants were collected. Extracellular ATP levels were quantified using an ATP Bioluminescence Assay Kit (Fisher Scientific), while HMGB1 concentrations were measured using an HMGB1 ELISA Kit (Chondrex).

BMDC Maturation

Bone marrow-derived dendritic cells (BMDCs) were obtained by sacrificing BALB/c mice (aged 6 to 8 weeks). The bone marrow was extracted from the femur and tibia using insulin syringes containing RPMI-1640. Sterile ACK buffer (Corning) was used to lyse the red blood cells, and the remaining cells were cultured in RPMI-1640 complete medium supplemented with 20 ng/mL recombinant mouse granulocyte-macrophage colony-stimulating factor (GM-CSF, R&D Systems) and 10 ng/mL recombinant murine interleukin-4 (IL-4, PeproTech). On day 4, the entire medium was discarded and replaced with a fresh, warm medium containing 20 ng/mL GM-CSF and 10 ng/mL IL-4. On day 6, the loosely attached cells in the semi-suspended state were collected by gentle pipetting, and the medium suspension containing these cells was designated as bone marrow-derived dendritic cells (BMDCs).

To determine DC maturation, CT26 cells were seeded in 6-well plates at a density of 2×10^5 cells/well and cultured overnight. MOF or R-MOF were added at an equivalent DBP concentration of 20 μM for 6 hours and irradiated with an LED light (630 nm, 80 mW/cm², 12.5 minutes). 24 hours later, the wells were pipetted up and down and transferred to 1.5mL Eppendorf (EP) tubes and centrifuged at 14000 rpm for 10 minutes at 4 ℃. The supernatants were then taken and co-incubated with BMDCs that were plated in 96-well plates at a density of 5×10^4 cells per well. After co-incubation for 24 hours, the culture supernatants were collected and analyzed using ELISA to measure IL-6 levels. The BMDCs were harvested to determine maturity ($CD11c^+/CD80^+/CD86^+$) via flow cytometry.

***In vitro* Phagocytosis**

Bone marrow-derived macrophages (BMDMs) were obtained by sacrificing a C57BL/6 mouse (aged 6 to 8 weeks). The bone marrow was extracted from the femur and tibia using insulin syringes containing DMEM. Sterile ACK buffer (Corning) was used to lyse the red blood cells, and the remaining cells were cultured in DMEM complete medium supplemented with 20 ng/mL recombinant mouse macrophage colony-stimulating factor (M-CSF, R&D Systems). On day 4, the entire medium was discarded and replaced with a fresh, warm medium containing 20 ng/mL M-CSF.

MC38 cells were cultured and seeded in 35 mm Glass bottom dishes (CellVis) at a density of 1×10^5 cells/well and cultured overnight. The cells were treated with PBS, MOF, or R-MOF (DBP concentration 20 μM) the following morning. 8 hours later, indicated cells were irradiated with LED light (630 nm, 100mW/cm^2) for 10 minutes. The cells were then washed with PBS and

stained with a CFSE cell division kit (BioLegend) for 30 minutes. The cells were then washed with PBS and 5×10^4 macrophages were added to each well. 24 hours later, the supernatants were removed, and the cells were washed with PBS. The cells were then fixed with 4% PFA (in PBS) for 10 minutes. The cells were washed again with PBS twice and then blocked with anti-CD16/32 antibody (clone 93, 1:100) for 20 minutes. The cells were then stained for F4/80-PerCp/Cy5.5 (1:100) for 30 minutes to stain for macrophages. The cells were then washed 3 times with PBS stained with Hoechst 33342 and examined with CLSM.

NF-kB Phosphorylation

CT26 cells were cultured in 6-well plates at a density of 1.5×10^5 cells/well and cultured overnight. The cells were then treated with PBS, MOF, or R-MOF (DBP concentration = 20μM) the next day. 8 hours later, the cells were irradiated with LED (630 nm, 100mW/cm^2) for 10 minutes. 14 hours later, the supernatant was collected and centrifuged at 14,000 rpm for 10 minutes. The supernatants were then added to 6-well plates that had been seeded with RAW246.7 cells at a density of 2×10^5 cells/well the night before. The RAW cells were then incubated for 15 minutes at 37°C and lysed. NF-kB was tested using a PathScan® Phospho-NF-κB p65 ELISA kit according to the manufacturer's protocol (Cell Signaling Technology).

S.1.5. *in vivo* Studies

***In vivo* Efficacy Studies**

The in vivo efficacy of R-MOF was tested on a subcutaneous CT26 tumor model and a subcutaneous MC38 tumor model. For the establishment of these models, 2×10^6 CT26 or MC38 cells were subcutaneously injected into the right flanks of BALB/c and C57BL/6 mice, respectively, on day 0. When the tumor volume reached around 50-80 mm^3 on day 7, the mice were randomized into several groups (n=6 for CT26 and n=5 for MC38). PBS, MOF, R-MOF, or MOF plus QP-R848 were injected intratumorally at an equivalent DBP dose of 0.5 μmol (or 50 ug R848 equivalent) on day 7. 8 hours later, the mice were anesthetized with 1.5% (V/V) isoflurane/O_2. The body of each mouse was covered with black cloth and the tumor area was irradiated with LED light (630nm, 100mW/cm^2) for 15 minutes. 48 hours after injection, blood was through the facial vein into heparin-coated centrifuge tubes. The blood was then centrifuged at 14,000 rpm for 10 minutes, and the supernatant was directly assayed with creatinine and aspartate transaminase (AST) kits per manufacturer protocol (Sigma Aldrich). Tumor volumes and body weights were monitored daily, and the volume was calculated as length×width^2/2. On day 22, the BALB/c mice were sacrificed, and the tumors were excised and sectioned for hematoxylin and eosin (H&E) staining and terminal deoxynucleotidyl transferase dUTP nick end labeling (TUNEL) staining. Major organs were harvested and sectioned for H&E staining. On day 26, the C57BL/6 mice were sacrificed, the tumors were harvested for T cell population analysis and the spleens of indicated mice were processed and used for an IFN-γ ELISPOT assay.

TGI was calculated with the following equation:

$$TGI = \frac{\overline{V_{PBS}} - \overline{V_i}}{\overline{V_{PBS}}} \times 100\%$$

Where $\overline{V_{PBS}}$ is the average tumor volume of the PBS group and $\overline{V_i}$ is the average tumor volume of the group of interest.

T-cell population analysis

MC38 tumor-bearing C57BL/6 mice (n=5) received treatment as above, and the tumors were harvested on day 6 for T-cell profiling by flow cytometry. The tumors were digested with 600 µL of DMEM + 10% FBS + 1 mg/mL collagenase I (Gibco) + 250 µg/mL collagenase IV (Gibco)+ 50 µg/mL DNase I (Sigma-Aldrich) cocktail at 37 °C for 45 minutes. The digests were neutralized with 4.4 mL complete DMEM medium and gently ground and filtered through sterile cell strainers (40 µm, Corning) to obtain single cell suspension (~10^7 cells/mL). The cell pellets were collected by centrifugation with 300 g for 10 minutes at 4 °C. For live staining, cells were washed with an ice-cold FACS buffer and stained first with LIVE/DEAD™ fixable yellow dead cell stain kit (ThermoFisher Scientific, 1:1000). The cells were then washed with FACS buffer, blocked by anti-CD16/32 antibody (clone 93, 1:100) at 4 °C for 1 hour, and stained with the fluorochrome-conjugated rat anti-mouse antibodies 1:200 (1:500 for CD45-BV421) at 4 °C for 30 minutes. The antibodies, conjugated dyes, and clone numbers were listed as follows: CD45-BV421, CD3ε-PE-efluor-610, CD4-APC/Cy7, and CD8α-PerCP/Cy5.5. All antibodies were from BioLegend. The cells were finally washed and resuspended in FACS buffer and analyzed on an LSR Fortessa 4-15 flow cytometer. Representative gating strategies are given in Fig. S6.

IFN-γ ELISPOT assay

A Multiscreen HTS-IP plate (Millipore Sigma) was activated by 70% ethanol, washed with PBS 4 times, coated with anti-mouse IFN-γ capture antibody (BD Biosciences) at 37 °C overnight, and blocked with sterile RPMI-1640 complete medium at room temperature for 2 hours. The spleens were harvested from the treated MC38 tumor-bearing C57BL/6 mice and were then gently ground and filtered through sterile cell strainers to afford single-cell suspensions. Red blood cells were then lysed by sterile ACK buffer (Corning), and splenocytes were counted and seeded in the plate at a density of 2 × 10^5 cells/well in RPMI-1640 full medium (5 mice in each group). MC38 tumor-associated KSPWFTTL (KSP) peptide was added to each well at a concentration of 10 µg/mL. The splenocytes were incubated at 37 °C for 48 hours and culture media were discarded. The plates were then washed and incubated with a biotinylated anti-IFN-γ detection antibody, streptavidin-HRP conjugate, and AEC substrate following the manufacturer's specification (BD Biosciences). The plate was air-dried and analyzed by a CTL ImmunoSpot® S6 Analyzer.

S1.6 Statistical analysis

Data are presented as mean ± standard deviation (SD) unless otherwise noted. Statistical analysis was performed on GraphPad Prism software using One-way Repeated Measures ANOVA method with Tukey's honest significance test. Statistical significance is represented as *P < 0.05, **P < 0.01, and ***P < 0.001.

S2 Supporting Figures and Tables

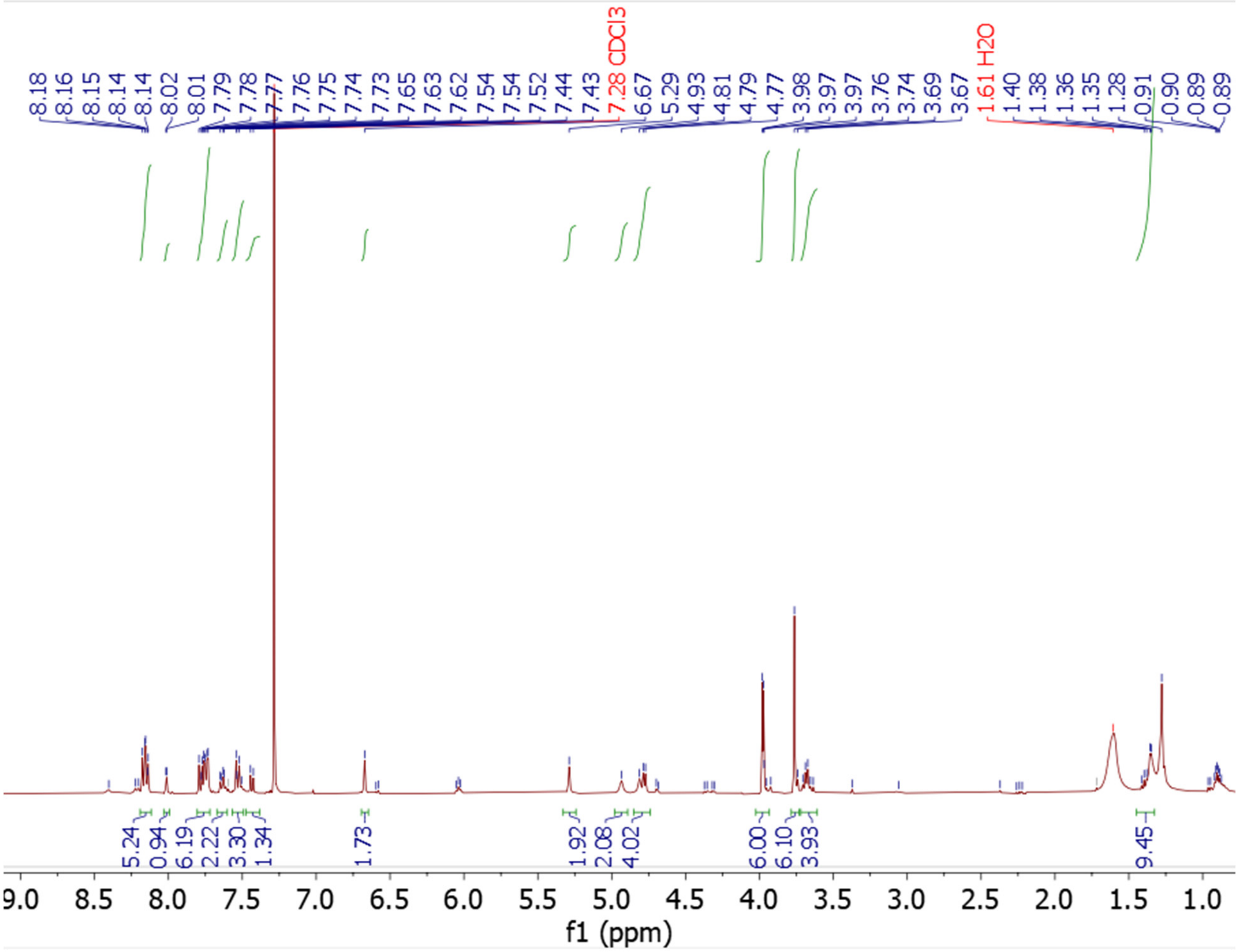

Figure S1. ^{1}H NMR spectrum of Me_2QP-R848.

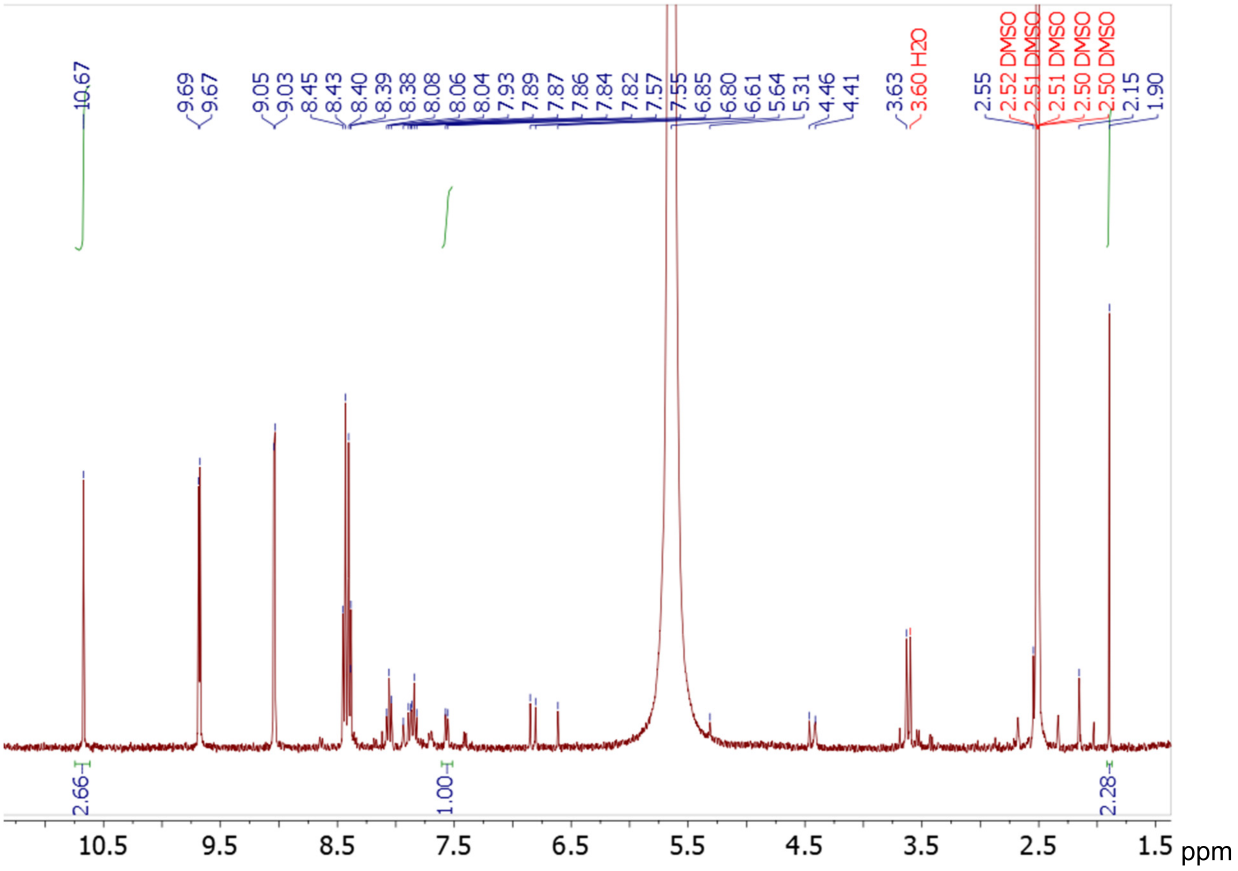

Figure S2. ^{1}H NMR spectrum of the digested MOF.

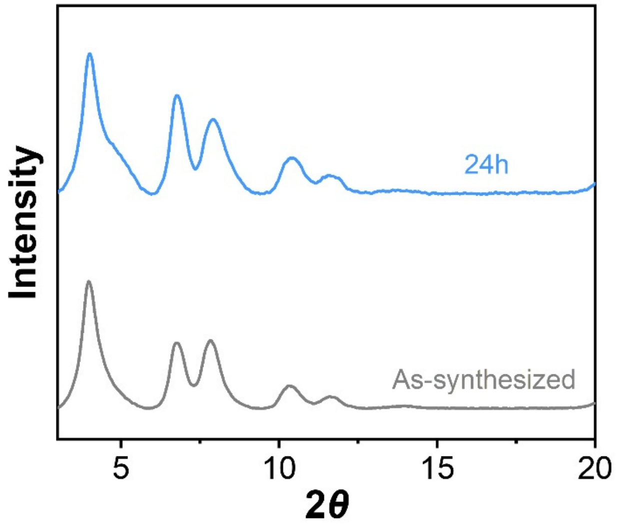

Figure S3. PXRD patterns of freshly prepared R-MOF and R-MOF after 24-hour incubation in PBS.

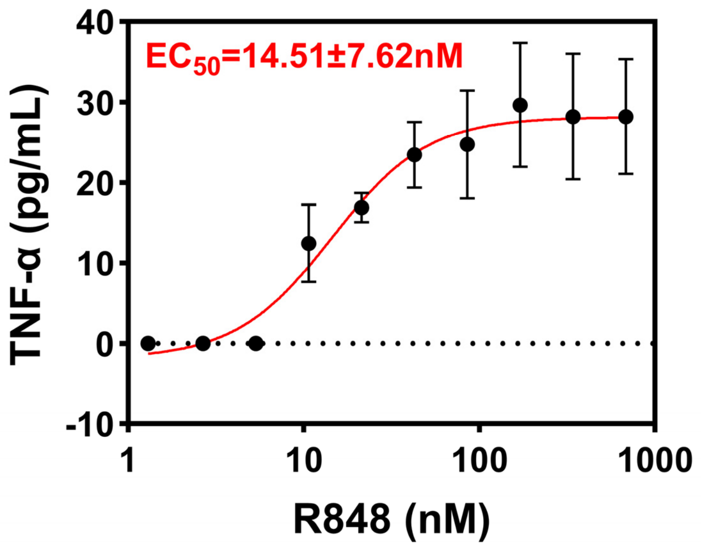

Figure S4. TNF-α secretion by RAW264.7 macrophages after incubation with different concentrations of R848 (n=3).

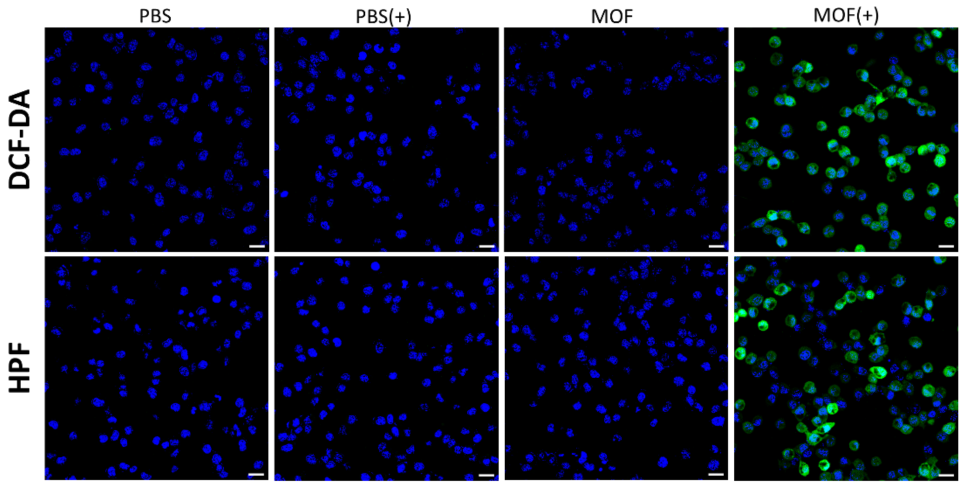

Figure S5. CLSM imaging of total ROS and hydroxyl radical detection in CT26 cells.

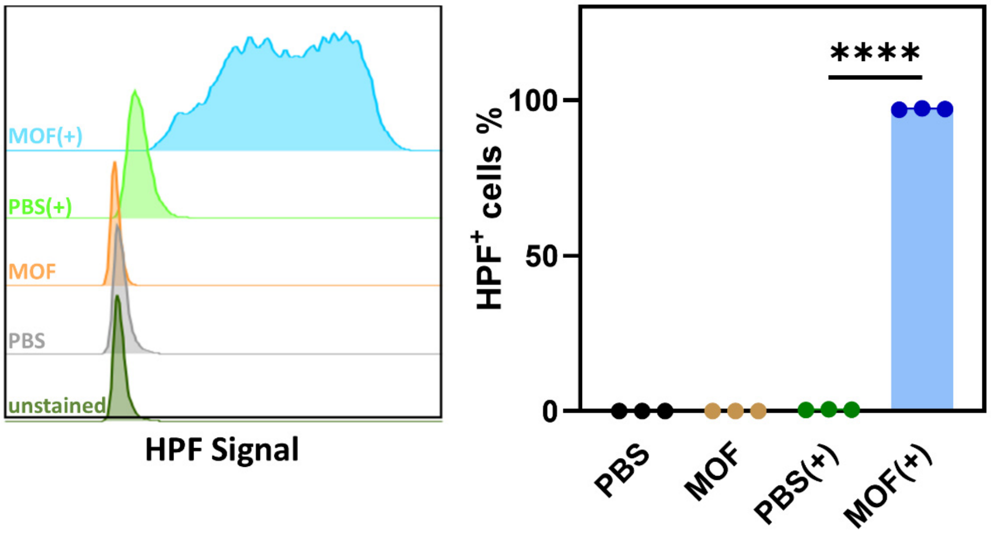

Figure S6. Flow cytometry analysis of hydroxyl radical generation in CT26 cells after different treatments (n = 3).

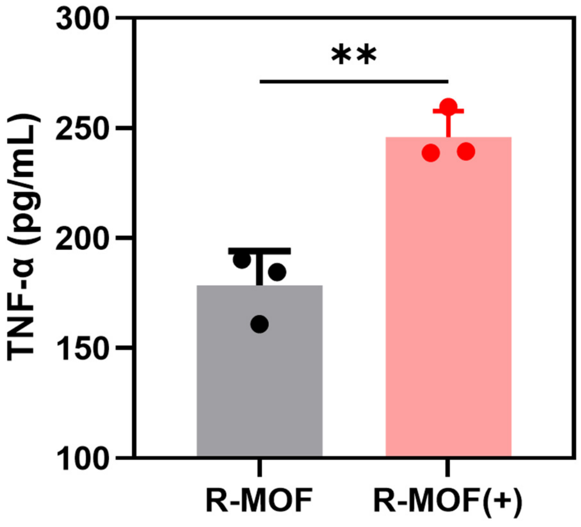

Figure S7. TNF-α release by BMDCs after exposure to R-MOF or R-MOF(+) at 10 μM DBP as analyzed with an ELISA assay (n = 3).

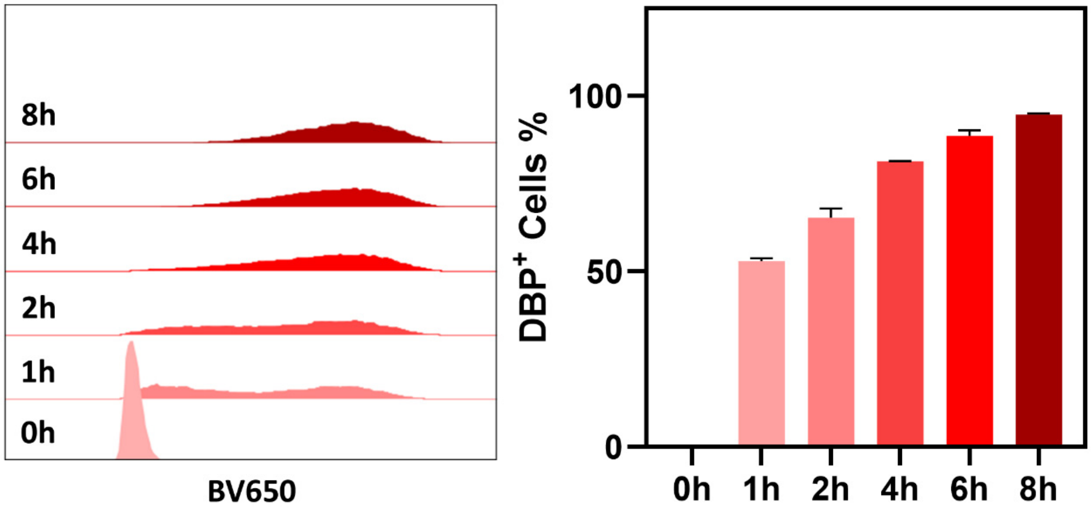

Figure S8. Time-dependent cellular uptake of MOF in CT26 cells (n = 3).

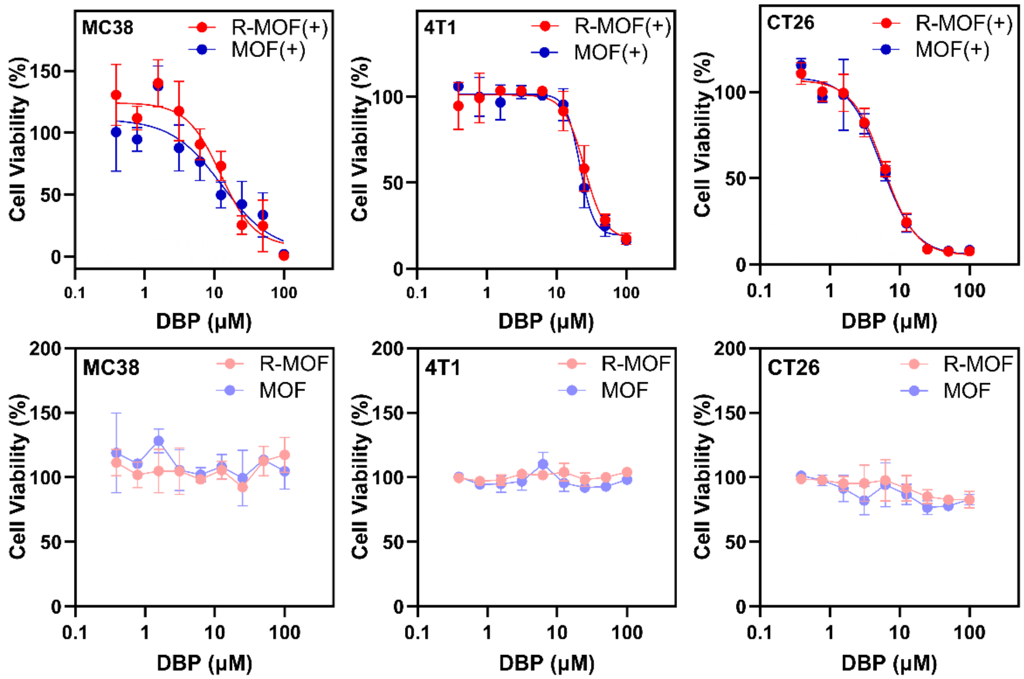

Figure S9. Cell viability studies of MOF and R-MOF on MC38, 4T1, and CT26 cells (n=3).

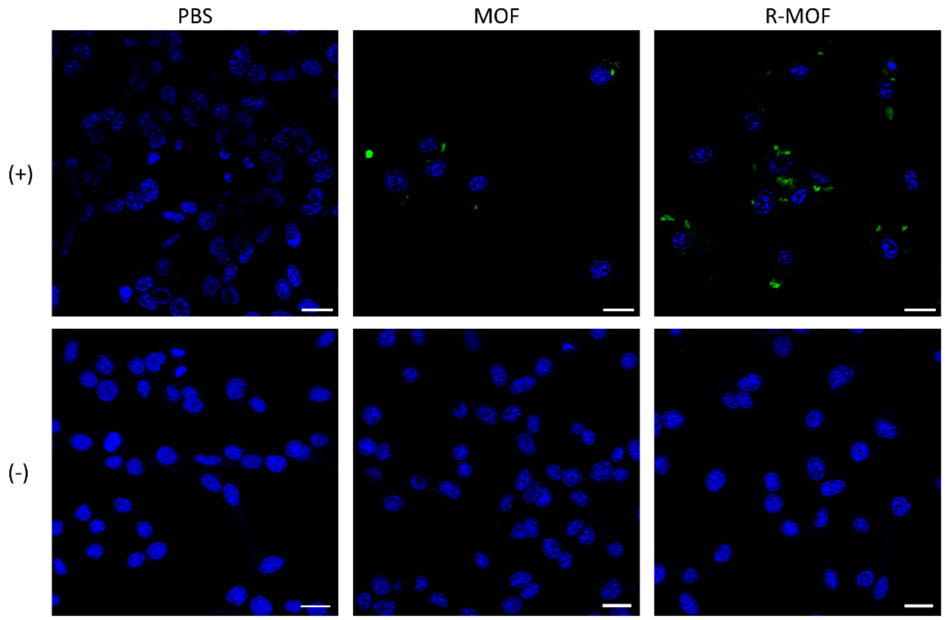

Figure S10. CLSM imaging of CRT after different treatments in CT26 cells.

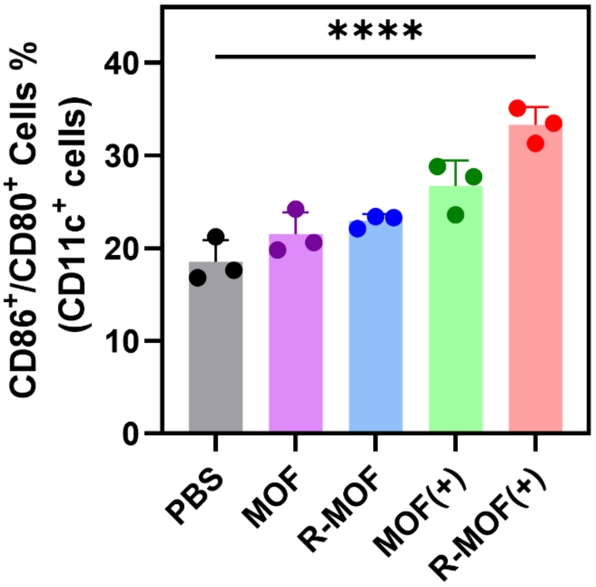

Figure S11. Bone marrow-derived dendritic cell maturation after exposure to the indicated treatments (n = 3).

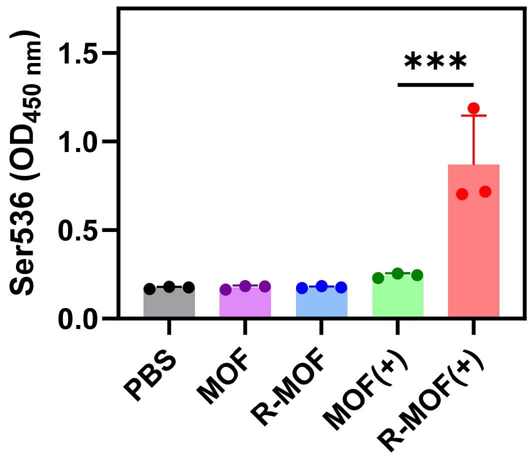

Figure S12. NF-kB Phosphorylation by RAW264.7 after exposure to CT26 cell supernatants following different treatments at 20 μM DBP, as analyzed with a Pathscan ELISA assay (n = 3).

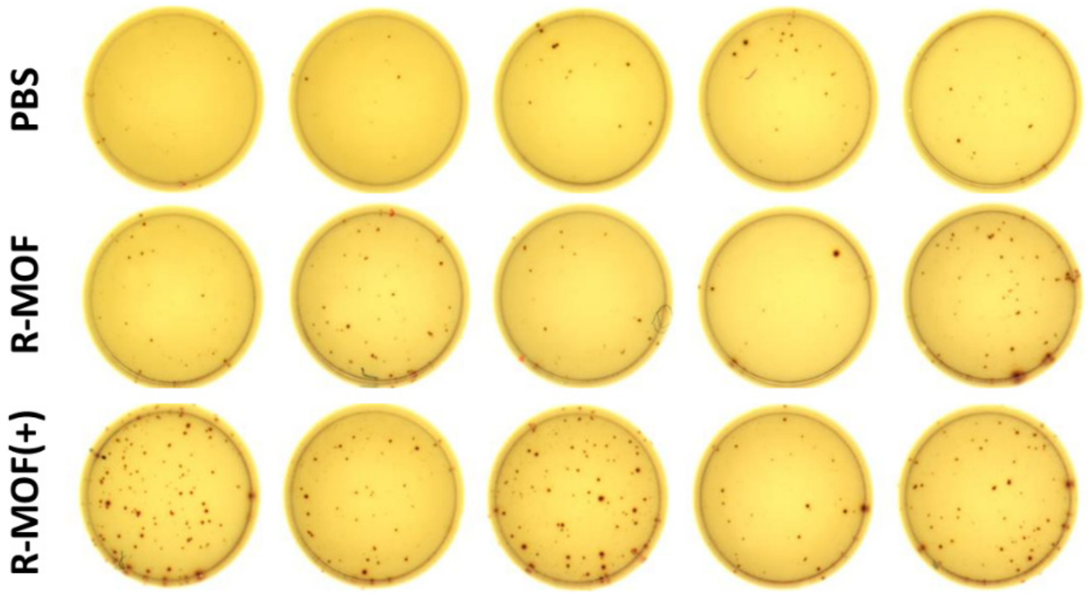

Figure S13. The photos of ELISPOT assay of splenocytes of MC38-bearing C57BL/6 mice after different treatments (n = 5).

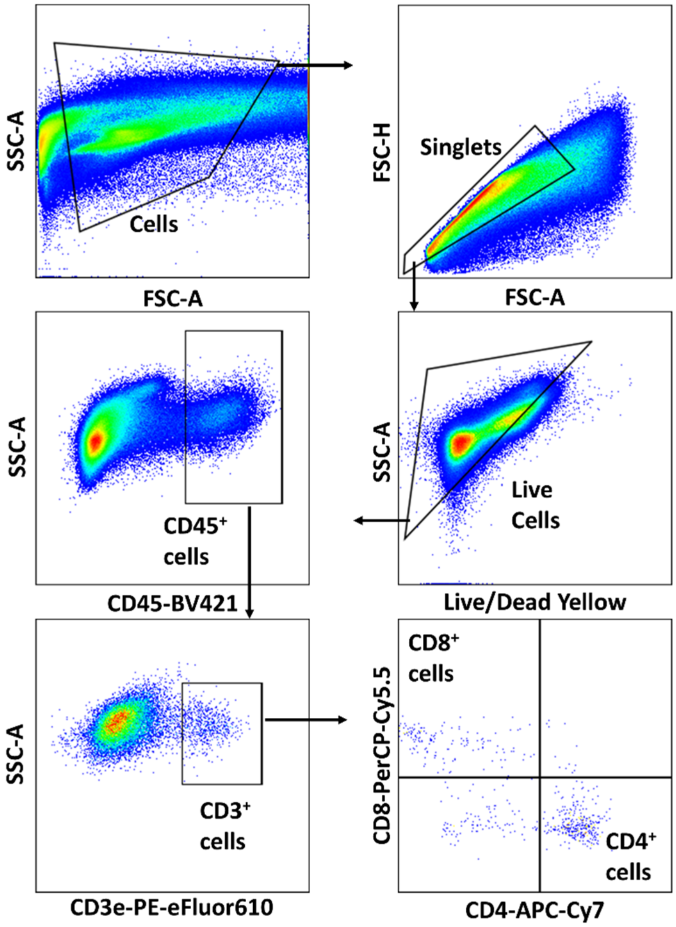

Figure S14. Gating strategy for late T cell responses in MC38-bearing C57BL/6 mice. T cells are defined as $CD45^+/CD3e^+$ cells, cytotoxic T cells are defined as $CD45^+/CD3e^+/CD8^+$ cells, and helper T cells are defined as $CD45^+/CD3e^+/CD4^+$ cells.

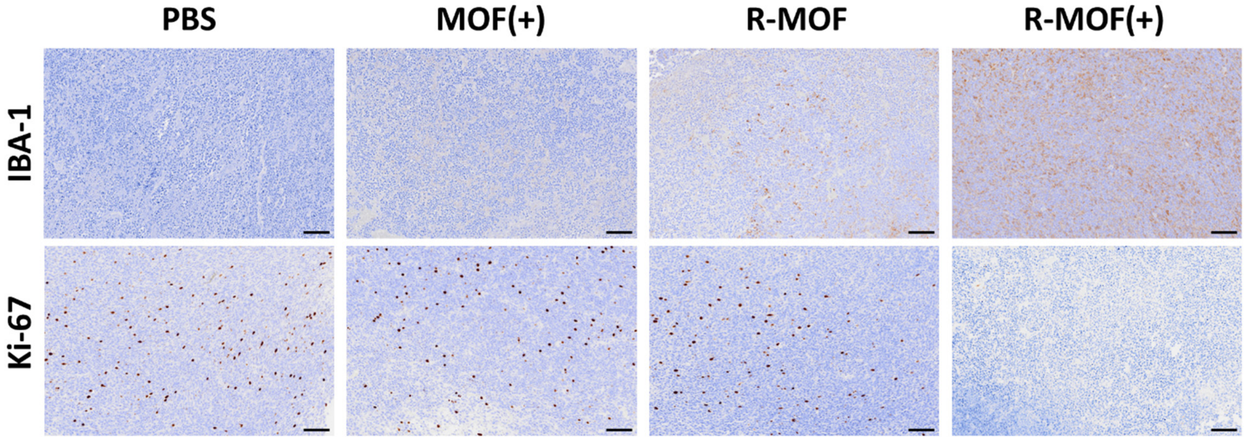

Figure S15. IBA 1 and Ki67 IHC staining of excised CT26 tumors at the endpoint. Scale bar: 100 μm.

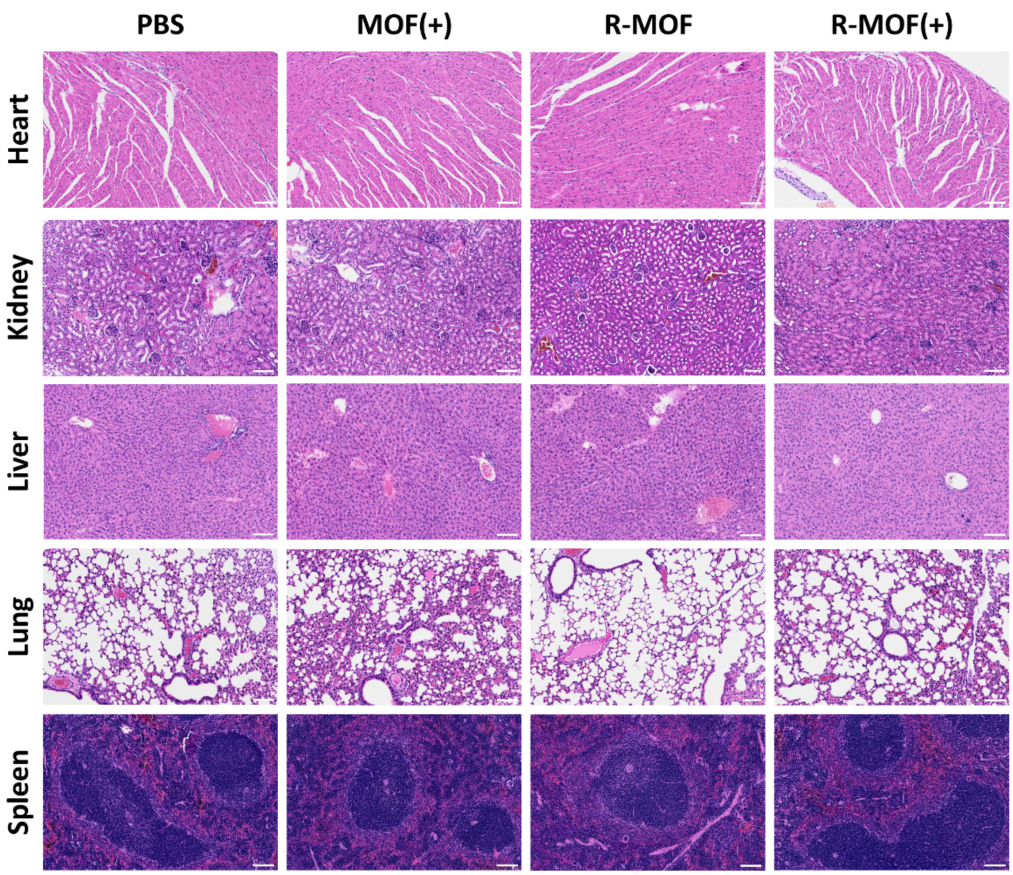

Figure S16. H&E staining of major organs excised from subcutaneous CT26 tumor-bearing mice at endpoint. Scale bar: 100 μm.

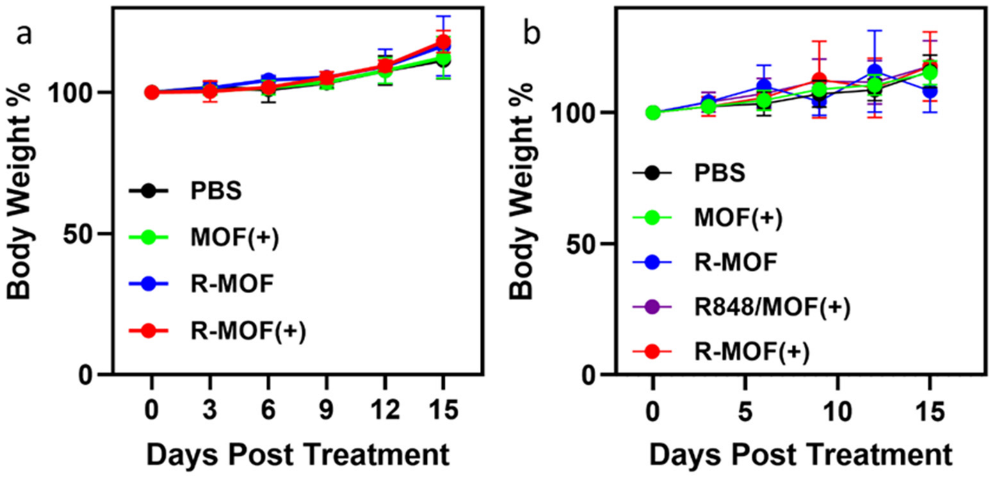

Figure S17. Body weight changes in (a) CT26 tumor-bearing BALB/c mice and (b) MC38 tumor-bearing C57BL/6 mice (n = 5) after different treatments.

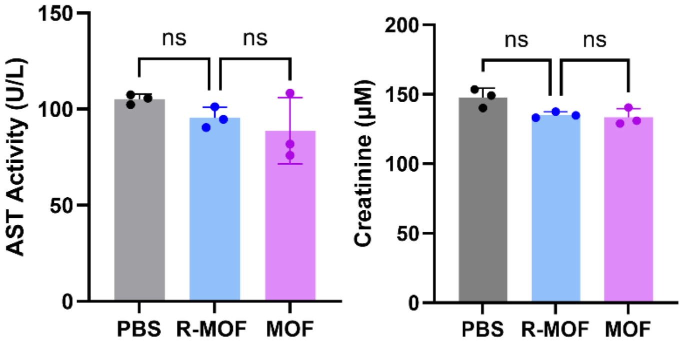

Figure S18. Blood aspartate aminotransferase (AST) activity and Creatinine concentrations of CT26 tumor-bearing BALB/c mice at 48 hours after different treatments (n=3).

References

[1] W. Zhen, Z. Xu, Y. Mao, C. McCleary, X. Jiang, R. R. Weichselbaum, W. Lin, *J. Am. Chem. Soc.* **2024**, *146*, 33149–33158.

[2] K. Lu, C. He, W. Lin, *J. Am. Chem. Soc.* **2014**, *136*, 16712–16715.
